# A Manual of Surgical Treatment

**Published:** 1901-09

**Authors:** 


### Reviews of Books.

**A Manual of Surgical Treatment.** By W. WATSON CHEYNE and F. F. BURGHARD. Part III. Pp. xvi., 305. Part IV. Pp. xx., 370. London: Longmans, Green and Co. 1900.

We have already referred to the general scope and excellent character of this work. Part III., which is devoted to diseases of bone, fractures, and amputations, is of the same standard of merit as the previous volumes, and is an eminently useful work to the surgeon and general practitioner.

We are glad to see that the fact that so-called "acute necrosis" may be recovered from without any necrosis at all, even when the periosteum is extensively stripped up from the bone, is clearly recognised by the authors. The free opening up of the medulla, in cases in which there is suppuration there, is recommended, and is of course clearly indicated ; but we are left very much in the dark as to the diagnosis of this condition, and as the authors very truly point out, if healthy medulla is opened, it may become infected from the pus between the periosteum and the bone. In the treatment of pyæmia following acute suppurative osteitis, if a thrombosed vein can be found, it is recommended that it should be ligated above the thrombus; but if no such thrombosed vein can be found, that the limb should be amputated. But why not ligate the main vein of the limb if the veins of the affected bone open into it, rather than amputate the limb, even if no clot can be found in it or its accessible branches? We hardly think amputation called for in cases of acute suppurative osteitis complicated by suppuration in the neighbouring joint, as we have seen several cases of recovery after free drainage of the joint, though it may be left rather stiff, and possibly the growth of the limb may be interfered with if the epiphyseal line should be attacked, as it probably would be in such a case.

We do not consider that the description of the displacement in fracture of the middle of the clavicle is very clear. There is no definite statement as to the position of the inner end of the outer fragment, only the shoulder and outer fragment are spoken of. In the treatment of this fracture we are glad to see an axillary pad is recommended as an addition to Sayer's method in all cases, so that the displacement inwards of the outer fragment is better corrected.

The desirability of using massage and early passive movements in fracture is, as we might expect, discussed, and we are advised not to adopt the possibly risky method of certain French surgeons in discarding the use of splints, but in many cases of fracture to temporarily remove splints and begin massage and passive movements very early. We must say that even this suggestion seems to us a little risky, as we should fear the possibility of imperfect union; but we feel sure that splints should be dispensed with at the earliest possible moment we can feel assured of union, and massage and passive movements very thoroughly used.

The reasons given for operation in all recent fractures of the patella are certainly very clear and powerful. The authors prefer the open method of operation, rather than the various subcutaneous ones.

The section of the book dealing with amputation is one of much value. The desirability of substituting irregular amputations for the set forms in cases of injury, so as to save every available inch of tissue, is strongly impressed on the reader, particularly in amputations about the hand and foot, and the need for stitching the divided tendons to the portion of the carpus and tarsus retained, is emphasised. It is pointed out how seldom such operations as Lisfranc's and Chopart's amputations can be performed in the living, though so frequently practised in classes of operative surgery on the dead body and given to students in examinations in operative surgery.

Part IV. is not only one of the largest but also one of the most valuable of the series, for it contains the chapters on tubercular disease of joints, a subject on which Mr. Watson Cheyne has long been regarded as an authority. We have, in fact, in these chapters much of the valuable information given in his work on the treatment of these affections, and we are glad to see some additional pictures are given.

The whole volume well maintains the high standard of excellence and utility which all the previous volumes possess. On account of its size, it demands a somewhat longer notice. We have not seen such useful illustrations of the methods employed in reducing dislocations in any other book. They are a great aid to the description of the manipulations; and the pictures of the anatomical relations of the head of the humerus in the various stages of Kocher's method of reducing dislocations of the shoulder are a valuable addition to the book.

We are glad to find a very good account of a disease about which nothing has been written in English text-books of surgery—so-called papillary synovitis of the knee-joint. It is very doubtful if it should be called synovitis at all, as its inflammatory nature is uncertain. We find great overgrowth of the processes of synovial membranes, so that the thickened fringes may be felt on palpation of the joint.

It is unfortunate that displacement of the semilunar cartilages should be twice treated of (p. 65 and again p. 224), especially as the instructions given for its treatment differ somewhat in the two places.

The question of the advisability of excising spina bifida is carefully considered, and the authors regard the operation as one to be preferred to the injection of Morton's fluid in many cases. The authors themselves allow that it is impossible to exclude the presence of nerve elements in the sac, and yet they make the presence or absence of such elements an indication for treatment. It is this difficulty in determining the absence of nerve elements in the sac which makes many surgeons hesitate to excise these tumours in cases in which the signs of such inclusion are absent. Dissecting out the nerves and returning them into the spinal canal may, as the authors say, be a very difficult matter: and this may be so even if a part of the sac-wall is left attached to the cord or nerves, and returned into the spinal canal with it. The novel proceeding of grafting the scapula or skull-bone of a rabbit over the opening in the arches is mentioned. In describing the treatment of spina bifida by the injection of Morton's fluid, in order to prevent leaking, we are advised to turn the infant on its face or side for a short time.

Surely our great object should be to prevent the passage of the fluid into the spinal canal.

We are not surprised to see that the authors do not consider laminectomy advisable in the ordinary form of the fracture-dislocation of the spine, but we are rather surprised to find it stated that "if the paralysis is partial, and judging from its gradual increase, and the irritative symptoms accompanying it at first, is probably due rather to pressure by blood-clot than by bony fragments, . . . it is well to perform laminectomy at once." If the pressure is from hæmorrhage, the probability is the hæmorrhage is in the cord itself, and the clot cannot be removed by operation. Moreover, if any displacement of the vertebræ is present, we doubt very much if the symptoms described would indicate hæmorrhage rather than bony compression. It seems to us that only the gradual increase would be suggestive of hæmorrhage. What are meant by "irritative symptoms," and why should they be present in hæmorrhage rather than in pressure on the cord and nerve roots? Then again, we entirely disagree with the authors when they say that signs of improvement in the paralysis are an indication for laminectomy. We suppose their idea is that if improvement occurs it is evident that the cord has not been hopelessly crushed at the moment when the displacement occurred, and, therefore, that the persisting pressure, rather than the initial crushing, is the cause of the paralysis, and may be removed by laminectomy. But surely, if improvement begins, it may encourage us to hope that recovery may take place without any laminectomy at all. We have lately had under our care a case of fracture-dislocation in the upper dorsal region (with quite distinct displacement) in which the power and sensation gradually returned, and the patient was able to walk about again, though with a certain amount of spastic rigidity in the lower limbs.

